# Multi-Dynamic-Multi-Echo-based MRI for the Pre-Surgical Determination of Sellar Tumor Consistency: a Quantitative Approach for Predicting Lesion Resectability

**DOI:** 10.1007/s00062-024-01407-1

**Published:** 2024-04-19

**Authors:** Mehmet Salih Yildirim, Victor Ulrich Schmidbauer, Alexander Micko, Lisa Lechner, Michael Weber, Julia Furtner, Stefan Wolfsberger, Intesar-Victoria Malla Houech, Anna Cho, Gregor Dovjak, Gregor Kasprian, Daniela Prayer, Wolfgang Marik

**Affiliations:** 1 Department of Biomedical Imaging and Image-guided Therapy, Medical University of Vienna, Waehringer Guertel 18–20, 1090 Vienna, Austria; 2 Department of Neurosurgery, Medical University of Graz, Auenbruggerplatz 29, 8036 Graz, Austria; 3 Department of Diagnostic Imaging, Medical University of Sofia, Sveti Georgi Sofiyski 1, 1431 Sofia, Bulgaria; 4 Department of Neurosurgery, Medical University of Vienna, Waehringer Guertel 18–20, 1090 Vienna, Austria

**Keywords:** Pituitary Adenoma, Multiparametric Magnetic Resonance Imaging, Prospective Studies, Reference Standards, Sellar Lesions

## Abstract

**Purpose:**

Pre-surgical information about tumor consistency could facilitate neurosurgical planning. This study used multi-dynamic-multi-echo (MDME)-based relaxometry for the quantitative determination of pituitary tumor consistency, with the aim of predicting lesion resectability.

**Methods:**

Seventy-two patients with suspected pituitary adenomas, who underwent preoperative 3 T MRI between January 2020 and January 2022, were included in this prospective study. Lesion-specific T1-/T2-relaxation times (T1R/T2R) and proton density (PD) metrics were determined. During surgery, data about tumor resectability were collected. A Receiver Operating Characteristic (ROC) curve analysis was performed to investigate the diagnostic performance (sensitivity/specificity) for discriminating between easy- and hard-to-remove by aspiration (eRAsp and hRAsp) lesions. A Mann-Whitney-U-test was done for group comparison.

**Results:**

A total of 65 participants (mean age, 54 years ± 15, 33 women) were enrolled in the quantitative analysis. Twenty-four lesions were classified as hRAsp, while 41 lesions were assessed as eRAsp. There were significant differences in T1R (hRAsp: 1221.0 ms ± 211.9; eRAsp: 1500.2 ms ± 496.4; *p* = 0.003) and T2R (hRAsp: 88.8 ms ± 14.5; eRAsp: 137.2 ms ± 166.6; *p* = 0.03) between both groups. The ROC analysis revealed an area under the curve of 0.72 (95% CI: 0.60–0.85) at *p* = 0.003 for T1R (cutoff value: 1248 ms; sensitivity/specificity: 78%/58%) and 0.66 (95% CI: 0.53–0.79) at *p* = 0.03 for T2R (cutoff value: 110 ms; sensitivity/specificity: 39%/96%).

**Conclusion:**

MDME-based relaxometry enables a non-invasive, pre-surgical characterization of lesion consistency and, therefore, provides a modality with which to predict tumor resectability.

**Supplementary Information:**

The online version of this article (10.1007/s00062-024-01407-1) contains supplementary material, which is available to authorized users.

## Introduction

Pituitary adenomas account for approximately 15% of primary intracranial tumors [1]. Although mostly benign, these lesions may show a tendency to affect anatomically adjacent structures in an invasive manner [1, 2]. Therefore, early neurosurgical interventions in certain cases are considered an effective treatment option that results in an auspicious clinical outcome [3]. Since the endoscopic, transnasal, transsphenoidal route has become the most widely used approach for surgical removal of pituitary tumors, a pre-surgical understanding of an individual’s anatomical complexity and lesion characteristics have become paramount [4].

While most pituitary adenomas can be easily extracted using this neurosurgical approach, 10–15% of these tumors are composed of fibrotic tissue, which are technically more demanding with regard to resection and require different neurosurgical techniques, as well as augmented resection equipment [5–7]. Therefore, pre-surgical knowledge of tumor consistency is crucial and will facilitate neurosurgical planning, which is key to improve postoperative clinical outcomes [2, 8–10]. However, modalities with which to assess pituitary adenoma consistency non-invasively prior to surgery are currently lacking [5, 11].

Multi-dynamic-multi-echo (MDME)-based imaging generates MRI contrasts based on tissue-specific properties (i.e., longitudinal and transverse relaxation times), acquired within a single scan of less than six minutes, by retrospective modulation of repetition time and echo time (i.e., image synthesis) [12–17]. Moreover, MDME-based sequences enable the quantification of tissue-specific relaxation time properties and proton density metrics [13, 16, 17]. While this novel modality has been investigated in various fields of clinical neuroradiology, studies that investigate lesion consistency using relaxometry are still scarce. Furthermore, only very few studies have focused on the applicability of contrast-enhanced MDME sequence acquisitions in a clinical setting [12, 18–20].

The aim of this prospective study was to investigate the feasibility of MDME-based MRI for the prediction of intraoperative resectability in a cohort of patients with suspected pituitary macroadenomas based on MRI evaluation. For this purpose, the lesion consistency was quantified by determining tissue-specific MR properties [T1-/T2-relaxation times (T1R/T2R) and proton density (PD) metrics] on non-enhanced, MDME-based imaging data. Lesion-specific MR properties of hard-to-remove tumors were compared to those considered easy-to-remove by aspiration during neurosurgery. In addition, relaxometry-based sensitivity/specificity characteristics and their respective cutoff values regarding lesion texture were analyzed. Furthermore, the feasibility of contrast-enhanced, MDME-based T1-weighted contrasts for the preoperative characterization of parasellar anatomy/pituitary adenomas was investigated. The results were compared to standard-of-reference T1-weighted sequences [Magnetization Prepared Rapid Acquisition Gradient Echo (MPRAGE) and Volumetric Interpolated Brain Examination (VIBE)].

## Materials and Methods

### Ethical Approval

The protocol of this prospective study was approved by the local ethics commission (Medical University of Vienna, Vienna, Austria) and performed in accordance with the Helsinki Declaration of 1975. All patients provided written, informed consent prior to MRI scanning and agreed to the scientific use of the acquired imaging data (EC-Nr.: 1549/2019).

### Study Cohort

**Fig. 1:**
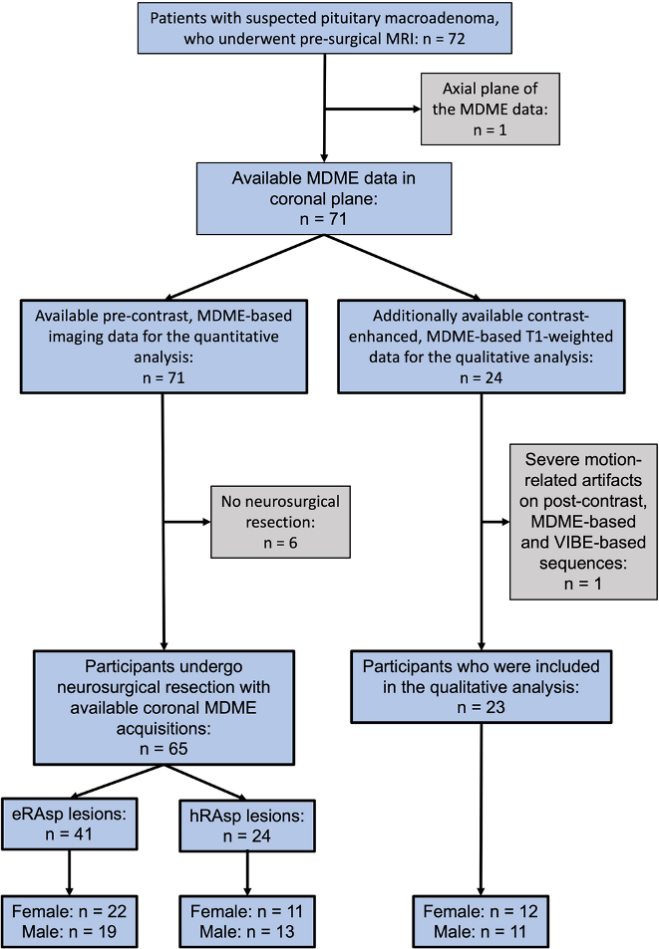
The flow diagram indicates how the study sample was derived. Patients in whom coronal multi-dynamic-multi-echo (MDME)-based imaging data were not available were excluded from this study (1/72). Furthermore, subjects who did not undergo neurosurgery were excluded from the quantitative analysis (6/72). Severe motion-related artifacts on post-contrast, MDME-based and Volumetric Interpolated Brain Examination (VIBE)-based imaging data were excluded from the qualitative analysis (1/72). In total, 65 participants [“easy-to-remove by aspiration” (eRAsp) = 41; “hard-to-remove by aspiration” (hRAsp) = 24] were included in the quantitative analysis, while 23 participants were enrolled in the qualitative assessment

Between January 2020 and January 2022, pre-surgical MRI was performed in 72 patients with a tumor in the pituitary region at the Department of Neuroradiology of a tertiary care hospital. All lesions have been morphologically delineated as pituitary macroadenomas based on MRI evaluation. Pituitary adenoma was histologically confirmed in 61 of the 72 patients (Table 1). Histological data were not available in 2 patients. Microadenoma patients were excluded from this investigation. Study sample characteristics are shown in Fig. 1 and Table 1.

**Table 1:** Characteristics of Included Participants

Demographics		Quantitative Assessment (*n* = 65)	Qualitative Assessment (*n* = 23)	Resectability ratio eRAsp/hRAsp (41/24)	P value*
*Sex*					0.54
Female		33	12	22/11	–
Male		32	11	19/13	–
*Mean age (y) at MRI*		54 ± 15	52 ± 18	–	0.22
*Lesion characteristics*^a^					
Chondrosarcoma^b^		–	*n* = 1	–	–
Corticotroph adenoma		*n* = 8	*n* = 2	7/1	–
Intrasellar meningioma^c^		*n* = 3	–	0/3	–
Gonadotroph adenoma		*n* = 25	*n* = 6	18/7	–
Craniopharyngioma		*n* = 1	–	0/1	–
Adenocarcinoma of the lung (Metastasis)		*n* = 1	–	0/1	–
Intrasellar plasmacytoma^d^		*n* = 1	–	0/1	–
LHC with pituitary infiltration		*n* = 1	–	0/1	–
Plurihormonal Pit-1-positive pituitary Adenoma		*n* = 1	–	1/0	–
Squamous cell carcinoma (Metastasis)		*n* = 1	–	0/1	–
Apoplectiform pituitary adenoma		*n* = 1	–	0/1	–
Lactotroph adenoma		*n* = 7	*n* = 5	6/1	–
Null cell adenoma		*n* = 9	*n* = 3	4/5	–
Somatotroph adenoma		*n* = 5	*n* = 3	4/1	–
Mammosomatotroph adenoma		*n* = 1	*n* = 1	1/0	–
*Data not available*		–	*n* = 2	–	–

^*^ Data were compared using the Pearson *χ*^2^ test for group homogeneity and the unpaired t‑test

^a^ According to World Health Organization (WHO) 2017 [32]

^b^ Chondrosarcoma presenting as pituitary adenoma [33]

^c^ Intrasellar meningioma mimicking pituitary adenoma [34]

^d^ Intrasellar plasmacytoma presenting as pituitary macro-adenoma [35]

*eRAsp* easy-to-remove by aspiration, *hRAsp* hard-to-remove by aspiration, *LHC* Langerhans cell histiocytosis, *MRI* Magnetic resonance imaging

### MR Data Acquisition

MRI was performed using the institutional routine imaging protocol for pre-neurosurgical planning (Table 2). Pre-contrast, MDME-based sequences (coronal plane) were added to the protocol. In 24 patients, post-contrast, MDME-based T1-weighted data were also available. The imaging data were acquired on a Siemens MAGNETOM Vida (3 T) MR system. Via two repeated acquisition phases [*phase a*, a slice-selective saturation pulse (flip angle: 120°) was applied to saturate one slice; *phase b*, a series of slice-selective refocusing pulses (flip angle: 180°) and a slice-selective excitation pulse (flip angle: 90°) were applied to generate spin echoes for another slice], the MDME sequence derived information about the physical MR properties of the tissue (T1-/T2-relaxation parameters and spin density) [13, 14, 21]. Data acquisition was performed at two different echo times (24 ms and 107 ms) following the 90° pulses, which were applied at four different saturation recovery times throughout the MDME scan cycle (i.e., eight spin echo acquisitions per scan cycle). Quantitative maps for the determination of lesion-specific properties were generated using the MDME post-processing software “SyMRI” (Synthetic MR AB, Linköping, Sweden; Version 11.2.9). Generation of parametric maps was performed within approximately ten seconds (per imaging data set), after transfer of MDME-based acquisitions from the MR scanner to a separate workstation for quantitative analysis. Contrast-enhanced, MDME-based T1-weighted data were generated automatically and retrieved on a *Picture Archiving and Communication System* (PACS) workstation for qualitative pre-surgical assessment.

**Table 2:** Magnetic Resonance Imaging Protocol

Sequence	Plane	Voxel size (mm^2^)	Slice thickness (mm)	Number of slices	FOV (mm)	TE (ms)	TR (ms)	AT
*TOF*	Tra	0.3 × 0.3	0.5	160	200	3.62	21	5:14
*MDME*	Cor	0.8 × 0.8	2.2	13	230	24; 107	4060	5:11
*Application of 0.1* *ml Gadobutrol (Gadovist®) per kg body weight*								
*MPRAGE*	Sag	0.9 × 0.9	0.9	192	240	2.32	2300	5:21
*CISS*	Cor	0.5 × 0.5	0.5	80	140	3.89	8.33	6:51
*VIBE*	Cor	0.7 × 0.7	0.7	144	210	2.58	7	5:21
*MDME*	Cor	0.8 × 0.8	2.2	13	230	24; 107	4060	5:11

*AT* Acquisition time, *CISS* Constructive interference in steady state, *FOV* Field-of-view, *MDME* Multi-dynamic-multi-echo, *MPRAGE* Magnetization Prepared Rapid Acquisition Gradient Echo, *TE* Echo time, *TR* Repetition time, *TOF* Time-of-flight angiography, *VIBE* Volumetric Interpolated Brain Examination

### Determination of Lesion-Specific MR Properties

**Fig. 2:**
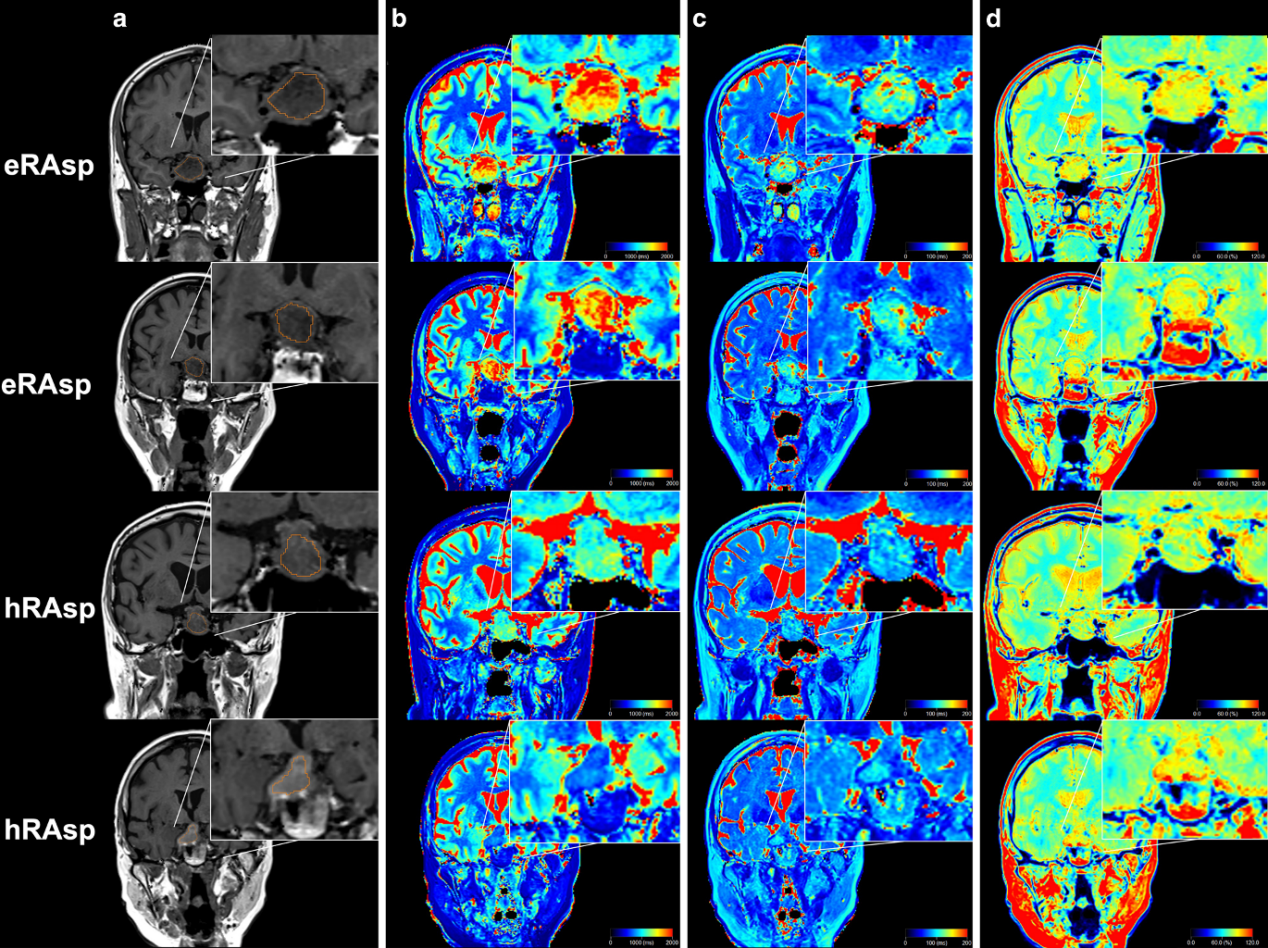
Non-enhanced, multi-dynamic-multi-echo (MDME)-based T1-weighted contrasts (**a**); quantitative T1-maps (**b**); quantitative T2-maps (**c**); and quantitative proton density (PD)-maps (**d**)are demonstrated (coronal plane). MR data of four different patients (*rows*) are presented: *1st row*, male subject, 34 years of age at the time of data acquisition (gonadotroph adenoma) [physical MR metrics based on the measurements of rater 1: 1741 ms (T1-relaxation time), 120 ms (T2-relaxation time), 85.3% (proton density)]; *2nd row*, female subject, 55 years of age at the time of data acquisition (null cell adenoma) [physical MR metrics based on the measurements of rater 1: 1709 ms (T1-relaxation time), 116 ms (T2-relaxation time), 85.7% (proton density)]; *3rd row*, male subject, 76 years of age at the time of data acquisition (null cell adenoma) [physical MR metrics based on the measurements of rater 1: 1165 ms (T1-relaxation time), 89 ms (T2-relaxation time), 80.5% (proton density)]; *4th row*, female subject, 65 years of age at the time of data acquisition (null cell adenoma) [physical MR metrics based on the measurements of rater 1: 865 ms (T1-relaxation time), 85 ms (T2-relaxation time), 71.8% (proton density)]. Region of interest (ROI) placement is demonstrated on the MDME-based T1-weighted contrasts (**a**). The colored bars indicate the T1-relaxation times [ms], T2-relaxation times [ms], and proton densities [%]. The relaxometric differences between “hard-to-remove by aspiration” (hRAsp) vs. “easy-to-remove by aspiration” (eRAsp) adenomas are well appreciated on the quantitative maps

Prior to the analysis, a critical visual review of the acquired MR data was performed by one neuroradiologist (W.M.) with 15 years of experience in neuroimaging. Highly artifact-degraded acquisitions were excluded. The lesion-specific properties [T1R (ms); T2R (ms); PD (%)] were measured by manually placing regions of interest (ROIs) at three different slices of the tumor on pre-contrast, “SyMRI”-generated parametric maps (Fig. 2). Units for T1R (spin-lattice-relaxation), T2R (spin-spin-relaxation), and PD (number of nuclei in the area being imaged) were adopted as provided by the “SyMRI”-based default software settings. ROI positioning was performed by two independent raters (M.S.Y. and L.L., each with one year of experience in pituitary MRI), who were blinded to intraoperative resectability assessments. In four cases, only two ROIs were drawn due to the limited availability of slices of the lesion. The average values of the quantitative properties were automatically computed, based on each single voxel value within the drawn ROI. Mean values, based on the three ROI measurements in each lesion, were calculated and used for further analysis (Supplementary Table 1).

### Assessment of Intraoperative Resectability of Sellar Lesions

The assessment of the intraoperative tumor extractability, via an endoscopic, transnasal, transsphenoidal approach, was assessed by the attending neurosurgeons (A.M. and S.W.) during surgery. In contrast to soft-constituted adenomas, tumors composed of fibrotic components are considered difficult to resect [5]. Therefore, the tumors were divided into two groups: “hard-to-remove by aspiration” (hRAsp) and “easy-to-remove by aspiration” (eRAsp). eRAsp lesions were freely removable by aspiration and only minimal use of curettage was required. Tumors that were difficult to aspirate, and which made curettage and successive mechanical debulking necessary, were assessed as hRAsp [5, 22].

### Qualitative Assessment of the Pituitary Region and Parasellar Anatomy

**Fig. 3:**
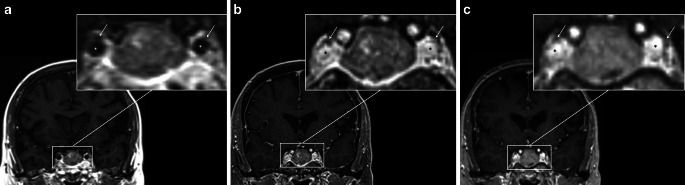
Contrast-enhanced, multi-dynamic-multi-echo-based (**a**); Volumetric Interpolated Brain Examination (VIBE)-based (**b**); and Magnetization Prepared Rapid Acquisition Gradient Echo (MPRAGE)-based (**c**) T1-weighted imaging data (*coronal plane*) depict parasellar anatomy comparably in a female patient, 22 years of age at the time of MRI (non-invasive, lactotroph adenoma). The left and right oculomotor nerve (*arrows*) is detectable on each sequence acquisition. Notice the differences in carotid artery enhancement (*asterisk*), while the pituitary adenoma enhances in a relatively stable manner, despite protocol-related delays (Table 2)

Qualitative assessments were performed on contrast-enhanced, MDME-based, VIBE-based, and MPRAGE-based T1-weighted data (coronal plane) (Fig. 3) by two independent senior neuroradiologists [J.F. (15 years of experience in neuroimaging) and W.M.]. To evaluate the pituitary region and parasellar anatomy, the following criteria were used [23]: the *Knosp* grade (for left/right cavernous sinus invasion) [23, 24]; parasellar invasiveness; normal gland position; detectability of the optic chiasm and left/right oculomotor nerve. The scoring criteria are explained in Supplementary Table 2 and Supplementary Fig. 1. Supplementary Fig. 2 shows the differences between pre- and post-contrast MDME-based acquisitions.

### Statistical Analyses

The statistical analyses were proposed and performed by one biomedical statistician with 30 years of experience (M.W.) using SPSS Statistics for Windows (Version 28.0; IBM Armonk, NY).

A *p*-value of *p* ≤ 0.05 was considered statistically significant. An intra-class correlation coefficient (ICC) analysis was used to assess the overall agreement between the mean values determined by both raters. ICC values ≥ 0.75 were considered strong agreements [25]. In case of strong correlations, the data based on the determinations performed by rater 1 were used for further analysis. A Mann-Whitney-U-test was performed to detect differences in T1R, T2R, and PD metrics between both groups (hRAsp vs. eRAsp). Receiver Operating Characteristic (ROC) curve analyses were performed to evaluate the predictive power of quantitative MRI metrics regarding the pre-surgical discrimination between hRAsp and eRAsp lesions. The Youden indices were calculated to determine optimal cutoff values for each quantitative metric.

The *Cohen’s Kappa coefficient* (κ) was used to detect: a) agreements between the observer’s qualitative assessments (on the basis of contrast-enhanced, MDME-based, VIBE-based, and MPRAGE-based T1-weighted data) (i.e., inter-rater agreement) and: b) concordances between the observer’s qualitative assessments based on the three different, post-contrast sequences (MDME vs. VIBE; MDME vs. MPRAGE; and VIBE vs. MPRAGE) (i.e., inter-sequence agreement). κ analyses were interpreted as proposed by Landis and Koch [26].

## Results

### Participant Characteristics

One of the 72 subjects was excluded from this study, due to the lack of coronal MDME sequence acquisitions. Furthermore, six of the 72 patients were excluded from the quantitative analysis of lesion consistency since they did not undergo a neurosurgical resection (Fig. 1). For the quantitative analysis, a total of 65 participants (mean age at data acquisition, 54 years ± 15) (female/male: 33/32) were enrolled in this study (Table 1). Based on the intraoperative assessment of tumor resectability, 24/65 (37%) tumors were classified as hRAsp, while 41/65 (63%) lesions were determined to be eRAsp. We found no evidence of differences between both groups in terms of age (unpaired t‑test: mean age, hRAsp: 57 years ± 13; eRAsp: 53 years ± 16; *p* = 0.22) or sex distribution (Pearson *χ*^2^ test: hRAsp, female/male: 11/13; eRAsp, female/male: 22/19; *p* = 0.54).

In 24 of the 72 patients, contrast-enhanced, MDME-based T1-weighted sequence acquisitions were also available. One of the 24 patients with contrast-enhanced acquisitions had to be excluded from the qualitative analysis, due to severe motion-related artifacts that were visible only on the contrast-enhanced, MDME-based and VIBE-based T1-weighted MR imaging data. Thus, for the qualitative sub-analysis of the pituitary region/parasellar anatomy, a total of 23 participants (mean age at data acquisition, 52 years ± 18) (female/male: 12/11) were included in this study (Table 1).

### ICC Analysis of the Determined MR Metrics

The ICC analysis revealed strong agreements between T1R [ICC: 0.99 (95% CI: 0.98–0.99), *p* < 0.001], T2R [ICC: 0.99 (95% CI: 0.98–0.99), *p* < 0.001], and the PD metrics [ICC: 0.85 (95% CI: 0.75–0.91, *p* < 0.001)] as determined by both raters.

### Predictability of Intraoperative Tumor Resectability

**Fig. 4:**
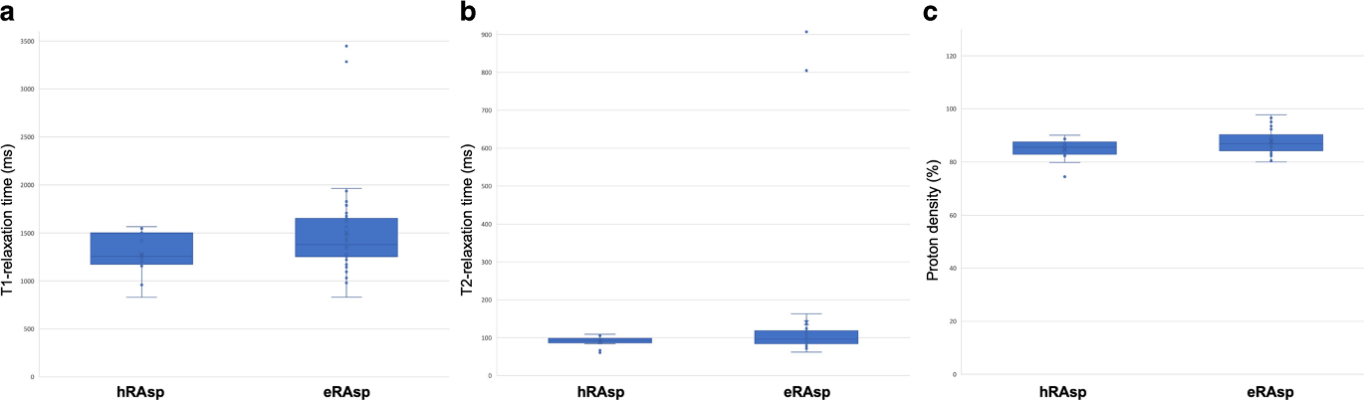
The boxplots show differences in T1-relaxation times (T1R) [ms] (**a**); T2-relaxation times (T2R) [ms] (**b**); and proton density (PD) values [%] (**c**), (measured by rater 1) between pituitary lesions that were hard- or easy-to-remove by aspiration (hRAsp vs. eRAsp). The *blue centered bar* indicates the mean values. Considerable resectability-related differences were found in T1R (**a**). Minor differences were observed in T2R (**b**)

Significant differences were found in T1R (hRAsp: 1221.0 ms ± 211.9; eRAsp: 1500.2 ms ± 496.4; *p* = 0.003) and T2R metrics (hRAsp: 88.8 ms ± 14.5; eRAsp: 137.2 ms ± 166.6; *p* = 0.03) between lesions classified as hRAsp vs. eRAsp. No evidence of differences was found in tumor resectability based on lesion-specific PD metrics (hRAsp: 85.6% ± 5.2; eRAsp: 87.7% ± 4.5; *p* = 0.11) (Fig. 4).

The ROC analysis revealed significant results for T1R, with an area under the curve (AUC) of 0.72 (95% CI: 0.60–0.85), *p* = 0.003; and T2R, with an AUC of 0.66 (95% CI: 0.53–0.79), *p* = 0.03. There was no evidence of significance for PD metrics, with an AUC of 0.62 (95% CI: 0.48–0.76), *p* = 0.11 (Table 3).

**Table 3:** Descriptive Statistics and Receiver Operating Characteristic Curve Analysis

MRI Parameter	Resectability	*n*	Mean (ms)	Std. Deviation (ms)	AUC	Asymptotic 95% CI	*p*-value*
*T1R*	**–**	65	1397.7	434.2	0.72	0.60–0.85	0.003
	eRAsp	41	1500.2	496.4	–	–	–
	hRAsp	24	1221.0	211.9	–	–	–
*T2R*	–	65	119.3	134.0	0.66	0.53–0.79	0.03
	eRAsp	41	137.2	166.6	–	–	–
	hRAsp	24	88.8	14.5	–	–	–
*PD*	–	65	86.9	4.8	0.62	0.48–0.76	0.11
	eRAsp	41	87.7	4.5	–	–	–
	hRAsp	21	85.6	5.2	–	–	–

^*^ Receiver Operating Characteristic curve analysis to investigate diagnostic performance and Mann-Whitney-U-test

*AUC* Area under the curve, *eRAsp* easy-to-remove by aspiration, *hRAsp* hard-to-remove by aspiration, *ICC* Intra-class correlation coefficient, *MRI* Magnetic resonance imaging, *PD* proton density, *T1R* T1-relaxation time, *T2R* T2-relaxation time

**Fig. 5:**
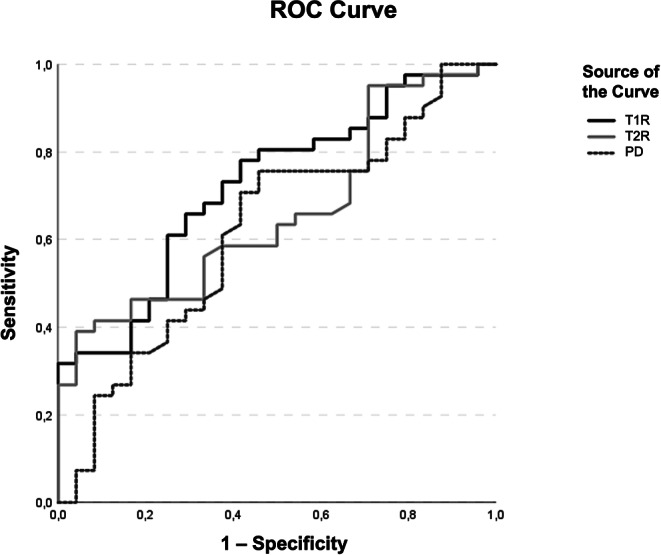
Depiction of diagnostic performance of T1-relaxation times (T1R) (*black curve*), T2-relaxation times (T2R) (*gray curve*), and proton densities (PD) (*dashed curve*) for the difficulty level of resectability depending on the corresponding cutoff values. The true-positive rate (sensitivity) (*y‑axis*) vs. the false-positive rate (1-specificity) *(x-axis*) is plotted at various threshold level settings. The most predictive results were achieved based on the T1R

For T1R, the sensitivity/specificity to predict easy tumor extractability was 78%/58% (Youden index: 0.364) at a cutoff value of 1248 ms. For T2R, the sensitivity/specificity to predict easy tumor resectability was 39%/96% (Youden index: 0.349) at a cutoff value of 110 ms. For PD, the sensitivity/specificity to predict easy tumor extractability was 76%/54% (Youden index: 0.298) at a cutoff value of 85% (Fig. 5).

### Qualitative Assessment of the Pituitary Region/Parasellar Anatomy Using Post-contrast MDME-Based, VIBE-Based, and MPRAGE-Based T1-Weighted MR Data

Inter-rater agreement between raters was almost perfect (κ > 0.8) for 6/7 (MDME/MPRAGE) and 5/7 (VIBE) evaluated aspects (Supplementary Table 2 and Supplementary Fig. 1). Agreement between raters was lowest (κ < 0.8) for the detectability of the left oculomotor nerve (MDME/VIBE/MPRAGE) and the optic chiasm (VIBE).

Inter-sequence agreement was almost perfect in 5/7 (MDME vs. MPRAGE) and 4/7 (MDME vs. VIBE/VIBE vs. MPRAGE) evaluated aspects. Agreement between sequences was lowest for the detectability of the left/right oculomotor nerve (MDME vs. VIBE/MDME vs. MPRAGE/VIBE vs. MPRAGE) and the optic chiasm (MDME vs. VIBE/VIBE vs. MPRAGE). Detailed information about inter-rater and inter-sequence agreements are given in Tables 4 and 5 and Supplementary Table 2.

**Table 4:** Inter-rater Agreement (κ)

Agreement (κ)	MDME	VIBE	MPRAGE
*Knosp grade—left*	0.82 (0.63–1)	0.94 (0.82–1)	0.88 (0.72–1)
*Knosp grade—right*	0.93 (0.79–1)	0.93 (0.79–1)	0.92 (0.78–1)
*Parasellar invasiveness*	0.81 (0.56–1)	0.82 (0.58–1)	1
*Normal gland position*	0.87 (0.71–1)	0.94 (0.81–1)	0.94 (0.81–1)
*Detectability of optic chiasm*	1	0.62 (0.13–1)	1
*Detectability of CN III—left*	0.63 (0.32–0.94)	0.59 (0.17–1)	0.70 (0.32–1)
*Detectability of CN III—right*	1	0.83 (0.52–1)	0.87 (0.60–1)

Numbers in parentheses are 95% confidence intervals

*CN* Cranial nerve, *MDME* Multi-dynamic-multi-echo, *MPRAGE* Magnetization Prepared Rapid Acquisition Gradient Echo, *VIBE* Volumetric Interpolated Brain Examination

**Table 5:** Inter-sequence Agreement (κ)

Agreement (κ)	MDME vs. VIBE		MDME vs. MPRAGE		VIBE vs. MPRAGE	
*Knosp grade—left*	0.94 (0.82–1)^a^	0.81 (0.63–1)^b^	1^a^	0.81 (0.63–1)^b^	0.94 (−0.42–0.38)^a^	1^b^
*Knosp grade—right*	1^a^	1^b^	0.92 (0.78–1)^a^	0.93 (0.79–1)^b^	0.92 (0.78–1)^a^	0.93 (0.79–1)^b^
*Parasellar invasiveness*	0.91 (0.73–1)^a^	0.91 (0.73–1)^b^	0.81 (0.56–1)^a^	0.81 (0.56–1)^b^	0.91 (0.73–1)^a^	0.91 (0.73–1)^b^
*Normal gland position*	0.94 (0.82–1)^a^	1^b^	0.94 (0.82–1)^a^	1^b^	1^a^	1^b^
*Detectability of optic chiasm*	0.33 (−0.25–0.91)^a^	0.78 (0.36–1)^b^	1^a^	1^b^	0.33 (−0.25–0.91)^a^	0.78 (0.36–1)^b^
*Detectability of CN III—left*	0.16 (−0.21–0.52)^a^	−0.01 (−0.40–0.38)^b^	0.34 (−0.01–0.70)^a^	0.33 (−0.11–0.77)^b^	−0.02 (−0.42–0.38)^a^	0.16 (−0.32–0.65)^b^
*Detectability of CN III—right*	0.22 (−0.16–0.59)^a^	0.13 (−0.26–0.52)^b^	0.48 (0.10–0.85)^a^	0.57 (0.22–0.92)^b^	0.40 (−0.07–0.87)^a^	0.40 (−0.09–0.88)^b^

^a^ Inter-sequence Agreement (κ) for Observer 1

^b^ Inter-sequence Agreement (κ) for Observer 2

Numbers in parentheses are 95% confidence intervals

*CN* Cranial nerve, *MDME* Multi-dynamic-multi-echo, *MPRAGE* Magnetization Prepared Rapid Acquisition Gradient Echo, *VIBE* Volumetric Interpolated Brain Examination

## Discussion

In this study, the feasibility of multi-dynamic-multi-echo (MDME)-based MRI for the quantitative and qualitative pre-neurosurgical characterization of lesions, which have been identified as pituitary macroadenomas on pre-surgical MR imaging, was investigated in a clinical setting. Relaxometry-based mapping enabled the non-invasive assessment of lesion consistency and, therefore, provided an easy-to-apply modality with which to predict intraoperative tumor resectability [T1-relaxation time (T1R): sensitivity/specificity 78%/58%; cutoff value of 1248 ms (AUC = 0.72) and T2-relaxation time (T2R): sensitivity/specificity 39%/96%; cutoff value of 110 ms (AUC = 0.66)]. Moreover, the results presented in this investigation suggest that contrast-enhanced, MDME-based T1-weighted contrasts enable robust pre-surgical evaluations similar to those provided by Magnetization Prepared Rapid Acquisition Gradient Echo (MPRAGE), standard-of-reference MR sequences.

Most pituitary adenomas are characterized by a soft lesion consistency, and, therefore, are easily removed using aspiration devices via minimally invasive, transnasal, transsphenoidal approaches. However, pituitary lesions that contain fibrous components may be difficult to extract using the aforementioned approach and require different neurosurgical techniques for removal [5, 7]. Pituitary adenomas with a fibrous component account for approximately 10–15% of sellar lesions [5–7]. These tumors are associated with lower total resection rates and higher risks of recurrence after surgery, which is accompanied by an unsatisfactory clinical outcome [2, 8–10]. Thus, *a priori* information about lesion texture may facilitate preoperative planning with the chance to improve post-surgical outcomes.

MDME-based imaging is considered a relatively novel MR approach, which excels because of the short examination time and the possibility to retrieve data for both quantitative and qualitative evaluations. Although there are several studies that have focused on the clinical applicability of this recent technology, there is a lack of information on the practicality of MDME-derived data for neurosurgical needs [12, 18–20]. Quantitative MR metrics are linked to tissue-specific properties. Primarily, the H_2_O/protein fraction of the tissue determines its relaxometric features, with increased relaxation times/PD metrics associated with higher water content and vice versa [27]. These considerations are in line with the presented data, since lesions classified as eRAsp revealed higher T1R, T2R, and PD metrics, while diametrically opposed results were observed for hRAsp tumors. Furthermore, our observations are in keeping with a previous study by Yamada et al., who demonstrated similar findings in a cohort of patients with meningiomas, based on a different MR mapping approach [28]. Thus, quantitative imaging modalities appear to provide robust biomarkers for the determination of lesion consistency, despite potential differences in mapping technology [29].

Nonetheless, currently, contrast-enhanced T1-weighted imaging, for the qualitative assessment, represents the mainstay prior to neurosurgery. The feasibility of post-contrast MDME-based T1-weighted contrasts was investigated using a scoring system that evaluated crucial anatomical aspects of pituitary surgery. MDME-derived data demonstrated non-inferiority to MPRAGE-based data. While almost excellent concordances were observed for nearly all evaluated aspects, inter-rater (MDME/MPRAGE) and inter-sequence (MDME vs. MPRAGE) agreement was lowest for the detectability of the left oculomotor nerve, most likely due to higher Knosp grades assigned for the left side. Interestingly, apart from lower inter-rater concordances for oculomotor nerve detection based on VIBE sequences, there was relatively low agreement for the detectability of the optic chiasm compared to MDME- and MPRAGE-based data, possibly explained by the fact that higher resolution MR acquisitions are more prone to motion-related artifacts [30]. Nonetheless, high-resolution MRI remains indispensable for pre-surgical planning [31].

MDME-based imaging provides the opportunity to supply multi-parametric characterizations of the tissue to be resected and enables investigators to retrieve reliable, post-contrast T1-weighted data for the anatomical assessment prior to surgery. Moreover, the presented approach provides the opportunity to reconstruct various MR contrasts based on a single scan, which may be of interest in a neurosurgical setting. However, this was beyond the scope of this work. Nonetheless, the investigated modality bears promising potential to aid in neurosurgical decision-making and may improve preoperative planning.

### Limitations

Several limitations require consideration. We included all pituitary tumors initially classified as macroadenomas based on MR imaging. Therefore, based on histology, various subtypes of macroadenomas were included. Furthermore, there were also other solitary tumor entities, mimicking macroadenomas, that were included in this study. The sample size for both quantitative and qualitative analyses was relatively small, which mandates the need for further studies to confirm our findings. Furthermore, there was a considerable delay between intravenous contrast agent administration and MDME-based sequence acquisitions for the qualitative sub-analysis. However, the acquired data proved sufficient to reliably study the applicability of MDME-based imaging data in a clinical setting. This study did not provide information on the feasibility of MDME-based imaging for the assessment of pituitary microadenomas since these require different imaging acquisition strategies [32]. Nonetheless, the relaxometric evaluation of pituitary microadenomas is of great interest and requires further consideration in the future.

## Conclusion

In summary, multi-dynamic-multi-echo (MDME)-based mapping represents a reliable method with which to predict the intraoperative resectability of sellar tumors by providing non-invasive biomarkers for lesion consistency. Moreover, synthetically generated, contrast-enhanced T1-weighted data approached a performance similar to that of the current standard-of-reference with regard to pre-surgical assessments of the pituitary region. Therefore, the presented imaging approach provides promising potential to aid in neurosurgical decision-making and to facilitate preoperative planning, which is key to improved neurosurgical performance and post-procedural outcomes. This investigation paves the way for multi-parametric, MDME-based mapping in clinical neurosurgery.

### Supplementary Information

**Supplementary Fig. 1: ***Knosp* grade characteristics are illustrated based on the descriptions by Knosp et al. and Micko et al. [23, 24]. a: *Knosp* grade 0: there is no extension of the medial carotid line (i); b: *Knosp* grade 1: there is extension of the medial line, but no extension of the intercarotid line (ii); c: *Knosp* grade 2: there is extension of the intercarotid line, but no extension of the lateral line (iii); d: *Knosp* grade 3A: there is extension of the lateral line superior to the intracavernous carotid artery; e: *Knosp* grade 3B: there is extension of the lateral line inferior to the intracavernous carotid artery; and f: the intracavernous carotid artery is totally surrounded

**Supplementary Fig. 2: **MRI data of a female subject (63 years of age at the time of data acquisition) with gonadotroph adenoma is presented. Non-enhanced (*1st row*) and post-contrast (*2nd ro*w) multi-dynamic-multi-echo (MDME)-based T1-weighted (a); T2-weighted (b); and proton density (PD)-weighted MR imaging data

**Supplementary Table 1: **Quantitative Magnetic Resonance Imaging Metrics Determined by Both Raters

**Supplementary Table 2: **Qualitative Assessments of the pituitary Region by both raters

## Data Availability

Data generated or analyzed during the study are available from the corresponding author by request.

## Abbreviations

AUC: Area under the curve
eRAsp: Easy-to-remove by aspiration
hRAsp: Hard-to-remove by aspiration
MDME: Multi-dynamic-multi-echo
MPRAGE: Magnetization Prepared Rapid Acquisition Gradient Echo
PD: Proton density
ROC: Receiver Operating Characteristic
T1R: T1-relaxation time
T2R: T2-relaxation time
VIBE: Volumetric Interpolated Brain Examination
