# Supplementary material for: Multi-Dynamic-Multi-Echo-based MRI for the Pre-Surgical Determination of Sellar Tumor Consistency: a Quantitative Approach for Predicting Lesion Resectability
Source: Clin Neuroradiol. 2024 Apr 19;34(3):663–73. doi: 10.1007/s00062-024-01407-1 (PMC11339083; doi:10.1007/s00062-024-01407-1)
Supplement: Supplementary file 1 — Supplementary Fig. 1: Knosp grade characteristics are illustrated based on the descriptions by Knosp et al. and Micko et al. [23, 24]. a: Knosp grade 0: there is no extension of the medial carotid line (i); b: Knosp grade 1: there is extension of the medial line, but no extension of the intercarotid line (ii); c: Knosp grade 2: there is extension of the intercarotid line, but no extension of the lateral line (iii); d: Knosp grade 3A: there is extension of the lateral line superior to the intracavernous carotid artery; e: Knosp grade 3B: there is extension of the lateral line inferior to the intracavernous carotid artery; and f: the intracavernous carotid artery is totally surrounded [file 62_2024_1407_MOESM1_ESM.docx]

**Supplementary Table 1: Quantitative Magnetic Resonance Imaging Metrics Determined by Both Raters**

| *n* = 65 | Sex | Histology | Resectability^a^ | Mean T1R – Rater 1 (ms) | Mean T2R – Rater 1 (ms) | Mean PD – Rater 1 (%) | Mean T1R – Rater 2 (ms) | Mean T2R – Rater 2 (ms) | Mean PD – Rater 2 (%) |
| --- | --- | --- | --- | --- | --- | --- | --- | --- | --- |
| 1 | M | Meningioma | 1 | 952.67 | 73.33 | 75.17 | 940.67 | 73.00 | 76.37 |
| 2 | M | Null cell adenoma | 1 | 1173.33 | 88.33 | 82.87 | 1278.00 | 92.67 | 82.67 |
| 3 | M | Null cell adenoma | 1 | 1547.33 | 95.33 | 87.57 | 1622.67 | 98.00 | 87.67 |
| 4 | M | Gonadotroph adenoma | 0 | 1279.33 | 79.67 | 83.93 | 1178.67 | 73.00 | 80.93 |
| 5 | M | Gonadotroph adenoma | 1 | 1272.67 | 98.33 | 79.80 | 1430.67 | 97.33 | 87.43 |
| 6 | F | Null cell adenoma | 0 | 1300.00 | 90.33 | 88.93 | 1288.00 | 89.67 | 88.53 |
| 7 | F | Gonadotroph adenoma | 0 | 1416.00 | 108.00 | 90.27 | 1359.33 | 103.67 | 91.80 |
| 8 | F | Langerhans cell histiocytosis | 1 | 927.67 | 68.67 | 88.90 | 948.67 | 66.67 | 86.27 |
| 9 | M | Gonadotroph adenoma | 0 | 1708.33 | 118.67 | 86.27 | 1702.33 | 118.33 | 86.80 |
| 10 | F | Null cell adenoma | 0 | 1788.00 | 136.00 | 93.47 | 1887.00 | 135.67 | 94.23 |
| 11 | M | Intrasellar plasmacytoma | 1 | 1027.33 | 88.67 | 84.97 | 1172.33 | 91.33 | 86.07 |
| 12 | F | Null cell adenoma | 0 | 1653.33 | 110.00 | 87.53 | 1681.33 | 113.33 | 87.80 |
| 13 | M | Gonadotroph adenoma | 0 | 1369.67 | 96.67 | 82.30 | 1302.00 | 89.33 | 80.80 |
| 14 | F | Lactotroph adenoma | 0 | 1096.00 | 79.67 | 96.57 | 1194.33 | 98.00 | 90.83 |
| 15 | M | Gonadotroph adenoma | 0 | 1221.00 | 77.33 | 83.17 | 1166.33 | 77.00 | 85.83 |
| 16 | F | Gonadotroph adenoma | 0 | 1282.67 | 96.33 | 89.00 | 1366.33 | 94.33 | 88.03 |
| 17 | M | Corticotroph adenoma | 0 | 1427.33 | 117.00 | 84.17 | 1457.67 | 129.33 | 84.47 |
| 18 | M | Gonadotroph adenoma | 0 | 1371.67 | 118.67 | 83.03 | 1585.67 | 168.00 | 85.27 |
| 19 | F | Null cell adenoma | 0 | 1254.33 | 78.67 | 83.97 | 1250.00 | 79.33 | 84.67 |
| 20 | F | Somatotroph adenoma | 1 | 1288.67 | 86.67 | 88.67 | 1399.67 | 88.00 | 85.73 |
| 21 | F | Null cell adenoma | 1 | 1155.67 | 67.00 | 82.30 | 1153.33 | 68.00 | 81.97 |
| 22 | M | Gonadotroph adenoma | 1 | 1206.00 | 89.67 | 89.43 | 1231.67 | 92.00 | 91.03 |
| 23 | F | Craniopharyngioma | 1 | 924.33 | 70.00 | 83.90 | 895.00 | 72.00 | 95.40 |
| 24 | F | Meningioma | 1 | 1171.33 | 105.33 | 95.97 | 1267.67 | 110.00 | 93.27 |
| 25 | F | Gonadotroph adenoma | 0 | 1563.00 | 113.00 | 86.90 | 1504.67 | 99.67 | 90.60 |
| 26 | F | Corticotroph adenoma | 0 | 1165.33 | 138.33 | 92.47 | 1350.00 | 185.00 | 81.10 |
| 27 | M | Lactotroph adenoma | 0 | 1480.00 | 102.50 | 95.35 | 1473.50 | 102.00 | 94.45 |
| 28 | F | Lactotroph adenoma | 0 | 1143.00 | 77.00 | 85.40 | 927.67 | 62.67 | 93.30 |
| 29 | M | Gonadotroph adenoma | 1 | 1209.00 | 92.33 | 84.57 | 1403.33 | 93.33 | 87.93 |
| 30 | F | Lactotroph adenoma | 1 | 959.00 | 60.50 | 90.05 | 955.50 | 70.00 | 99.40 |
| 31 | M | Corticotroph adenoma | 0 | 1271.00 | 82.00 | 86.27 | 1070.33 | 81.67 | 88.37 |
| 32 | F | Lactotroph adenoma | 0 | 1441.00 | 96.33 | 89.90 | 1392.67 | 94.67 | 91.43 |
| 33 | M | Somatotroph adenoma | 0 | 1826.33 | 112.67 | 92.30 | 1709.33 | 108.00 | 90.67 |
| 34 | F | Lactotroph adenoma | 0 | 1935.00 | 163.33 | 97.73 | 2058.33 | 194.67 | 98.37 |
| 35 | M | Gonadotroph adenoma | 1 | 1243.67 | 91.67 | 87.03 | 1340.67 | 92.33 | 91.30 |
| 36 | F | Mammosomatotroph adenoma | 0 | 1272.33 | 80.67 | 86.13 | 1290.00 | 86.00 | 86.07 |
| 37 | F | Null cell adenoma | 1 | 829.33 | 92.67 | 74.43 | 865.00 | 82.33 | 74.63 |
| 38 | M | Null cell adenoma | 1 | 1422.33 | 99.33 | 85.23 | 1578.00 | 148.67 | 97.93 |
| 39 | F | Gonadotroph adenoma | 0 | 1654.33 | 122.67 | 85.47 | 1754.00 | 121.00 | 87.37 |
| 40 | F | Lactotroph adenoma | 0 | 1677.50 | 111.00 | 87.65 | 1649.00 | 108.00 | 89.60 |
| 41 | M | Gonadotroph adenoma | 0 | 1171.67 | 85.67 | 90.40 | 1158.67 | 83.00 | 91.30 |
| 42 | M | Somatotroph adenoma | 0 | 3284.67 | 804.67 | 95.07 | 3249.00 | 750.67 | 92.63 |
| 43 | F | Gonadotroph adenoma | 0 | 1031.33 | 71.67 | 80.50 | 1015.33 | 74.00 | 80.93 |
| 44 | M | Gonadotroph adenoma | 0 | 1963.00 | 143.33 | 87.63 | 2018.33 | 144.00 | 89.63 |
| 45 | F | Corticotroph adenoma | 0 | 831.00 | 62.00 | 81.05 | 866.00 | 67.33 | 78.17 |
| 46 | M | Gonadotroph adenoma | 1 | 1255.67 | 84.00 | 84.00 | 1044.00 | 79.33 | 82.23 |
| 47 | M | Corticotroph adenoma | 1 | 1566.67 | 105.33 | 87.17 | 1670.33 | 130.33 | 85.30 |
| 48 | F | Gonadotroph adenoma | 0 | 1253.00 | 85.67 | 82.40 | 1318.33 | 91.33 | 82.67 |
| 49 | M | Corticotroph adenoma | 0 | 1343.33 | 95.33 | 85.83 | 1430.00 | 98.33 | 86.07 |
| 50 | M | Gonadotroph adenoma | 0 | 1624.00 | 114.00 | 87.03 | 1621.67 | 111.33 | 86.43 |
| 51 | M | Gonadotroph adenoma | 0 | 1567.00 | 124.33 | 85.87 | 1645.67 | 132.33 | 85.83 |
| 52 | F | Corticotroph adenoma | 0 | 1380.00 | 86.67 | 86.23 | 1329.00 | 84.00 | 84.73 |
| 53 | M | Somatotroph adenoma | 0 | 3448.67 | 907.00 | 97.13 | 3298.00 | 724.00 | 96.23 |
| 54 | M | Plurihormonal Pit-1-positive pituitary adenoma | 0 | 1495.33 | 96.00 | 87.07 | 1544.00 | 99.33 | 87.67 |
| 55 | F | Adenocarcinoma of the lung | 1 | 1174.67 | 75.33 | 84.37 | 1160.67 | 74.00 | 84.63 |
| 56 | M | Gonadotroph adenoma | 0 | 1283.67 | 91.00 | 86.03 | 1407.00 | 104.33 | 84.33 |
| 57 | F | Squamous cell carcinoma | 1 | 1479.00 | 99.67 | 84.80 | 1363.67 | 90.00 | 84.70 |
| 58 | M | Apoplectiform pituitary adenoma | 1 | 1211.33 | 115.33 | 97.93 | 1258.33 | 123.67 | 96.67 |
| 59 | F | Gonadotroph adenoma | 0 | 1176.67 | 89.00 | 86.60 | 1234.33 | 89.67 | 92.93 |
| 60 | M | Meningioma | 1 | 1281.00 | 76.00 | 84.50 | 1298.00 | 77.00 | 85.07 |
| 61 | M | Somatotroph adenoma | 0 | 980.00 | 76.67 | 80.00 | 978.00 | 77.00 | 79.50 |
| 62 | F | Gonadotroph adenoma | 0 | 1500.33 | 102.00 | 89.33 | 1586.00 | 101.00 | 89.67 |
| 63 | F | Gonadotroph adenoma | 1 | 1502.33 | 109.33 | 85.57 | 1570.00 | 106.67 | 88.60 |
| 64 | F | Corticotroph adenoma | 0 | 1578.33 | 84.33 | 88.67 | 1516.00 | 84.33 | 89.43 |
| 65 | F | Gonadotroph adenoma | 1 | 1522.33 | 98.00 | 86.13 | 1579.67 | 99.33 | 88.30 |

^a^ 0: easy-to-remove by aspiration; 1: hard-to-remove by aspiration

F: Female

M: Male

PD: Proton density

T1R: T1-relaxation time

T2R: T2-relaxation time
