# Supplementary material for: Multi-Dynamic-Multi-Echo-based MRI for the Pre-Surgical Determination of Sellar Tumor Consistency: a Quantitative Approach for Predicting Lesion Resectability
Source: Clin Neuroradiol. 2024 Apr 19;34(3):663–73. doi: 10.1007/s00062-024-01407-1 (PMC11339083; doi:10.1007/s00062-024-01407-1)
Supplement: Supplementary file 2 — Supplementary Fig. 2: MRI data of a female subject (63 years of age at the time of data acquisition) with gonadotroph adenoma is presented. Non-enhanced (1st row) and post-contrast (2nd row) multi-dynamic-multi-echo (MDME)-based T1-weighted (a); T2-weighted (b); and proton density (PD)-weighted MR imaging data [file 62_2024_1407_MOESM2_ESM.docx]

**Supplementary Table 2: Qualitative Assessments of the Pituitary Region**

| *n*=23 | *Knosp* grade – left^a^ | | | *Knosp* grade – right^a^ | | | Invasiveness^b^ | | | Normal gland^c^ position^c^ | | | Optic chiasm^d^ | | | CN III – left^d^ | | | CN III – right^d^ | | |
| --- | --- | --- | --- | --- | --- | --- | --- | --- | --- | --- | --- | --- | --- | --- | --- | --- | --- | --- | --- | --- | --- |
|  | **a** | **b** | **c** | **a** | **b** | **c** | **a** | **b** | **c** | **a** | **b** | **c** | **a** | **b** | **c** | **a** | **b** | **c** | **a** | **b** | **c** |
| 1 | 3^e^; 3^f^ | 3^e^; 3^f^ | 3^e^; 3^f^ | 1^e^; 1^f^ | 1^e^; 1^f^ | 1^e^; 1^f^ | 1^e^; 1^f^ | 1^e^; 1^f^ | 1^e^; 1^f^ | 0^e^; 0^f^ | 0^e^; 0^f^ | 0^e^; 0^f^ | 1^e^; 1^f^ | 1^e^; 1^f^ | 1^e^; 1^f^ | 0^e^; 1^f^ | 0^e^; 1^f^ | 0^e^; 1^f^ | 1^e^; 1^f^ | 1^e^; 1^f^ | 1^e^; 1^f^ |
| 2 | 1^e^; 1^f^ | 1^e^; 1^f^ | 1^e^; 1^f^ | 1^e^; 1^f^ | 1^e^; 1^f^ | 1^e^; 1^f^ | 0^e^; 0^f^ | 0^e^; 0^f^ | 0^e^; 0^f^ | 0^e^; 0^f^ | 0^e^; 0^f^ | 0^e^; 0^f^ | 1^e^; 1^f^ | 1^e^; 1^f^ | 1^e^; 1^f^ | 1^e^; 1^f^ | 1^e^; 1^f^ | 1^e^; 1^f^ | 1^e^; 1^f^ | 1^e^; 1^f^ | 1^e^; 1^f^ |
| 3 | 0^e^; 0^f^ | 0^e^; 0^f^ | 0^e^; 0^f^ | 1^e^; 1^f^ | 1^e^; 1^f^ | 1^e^; 1^f^ | 1^e^; 0^f^ | 1^e^; 0^f^ | 0^e^; 0^f^ | 2^e^; 0^f^ | 2^e^; 0^f^ | 2^e^; 0^f^ | 1^e^; 1^f^ | 1^e^; 1^f^ | 1^e^; 1^f^ | 1^e^; 1^f^ | 1^e^; 1^f^ | 1^e^; 1^f^ | 1^e^; 1^f^ | 1^e^; 1^f^ | 1^e^; 1^f^ |
| 4 | 0^e^; 0^f^ | 0^e^; 0^f^ | 0^e^; 0^f^ | 0^e^; 0^f^ | 0^e^; 0^f^ | 0^e^; 0^f^ | 0^e^; 0^f^ | 0^e^; 0^f^ | 0^e^; 0^f^ | 1^e^; 1^f^ | 1^e^; 1^f^ | 1^e^; 1^f^ | 1^e^; 1^f^ | 1^e^; 1^f^ | 1^e^; 1^f^ | 0^e^; 0^f^ | 1^e^; 1^f^ | 0^e^; 0^f^ | 0^e^; 0^f^ | 1^e^; 1^f^ | 0^e^; 0^f^ |
| 5 | 0^e^; 0^f^ | 0^e^; 0^f^ | 0^e^; 0^f^ | 0^e^; 0^f^ | 0^e^; 0^f^ | 0^e^; 0^f^ | 0^e^; 0^f^ | 0^e^; 0^f^ | 0^e^; 0^f^ | 1^e^; 1^f^ | 1^e^; 1^f^ | 1^e^; 1^f^ | 1^e^; 1^f^ | 1^e^; 1^f^ | 1^e^; 1^f^ | 1^e^; 1^f^ | 1^e^; 1^f^ | 0^e^; 1^f^ | 1^e^; 1^f^ | 1^e^; 1^f^ | 0^e^; 1^f^ |
| 6 | 0^e^; 0^f^ | 0^e^; 0^f^ | 0^e^; 0^f^ | 1^e^; 1^f^ | 1^e^; 1^f^ | 1^e^; 1^f^ | 0^e^; 0^f^ | 1^e^; 1^f^ | 1^e^; 1^f^ | 2^e^; 2^f^ | 2^e^; 2^f^ | 2^e^; 2^f^ | 1^e^; 1^f^ | 1^e^; 1^f^ | 1^e^; 1^f^ | 1^e^; 1^f^ | 1^e^; 1^f^ | 1^e^; 1^f^ | 1^e^; 1^f^ | 1^e^; 1^f^ | 1^e^; 1^f^ |
| 7 | 0^e^; 0^f^ | 0^e^; 0^f^ | 0^e^; 0^f^ | 0^e^; 0^f^ | 0^e^; 0^f^ | 1^e^; 0^f^ | 0^e^; 0^f^ | 0^e^; 0^f^ | 0^e^; 0^f^ | 1^e^; 1^f^ | 1^e^; 1^f^ | 1^e^; 1^f^ | 1^e^; 1^f^ | 1^e^; 1^f^ | 1^e^; 1^f^ | 1^e^; 1^f^ | 1^e^; 1^f^ | 1^e^; 1^f^ | 1^e^; 1^f^ | 1^e^; 1^f^ | 1^e^; 1^f^ |
| 8 | 2^e^; 1^f^ | 2^e^; 2^f^ | 2^e^; 2^f^ | 0^e^; 0^f^ | 0^e^; 0^f^ | 0^e^; 0^f^ | 1^e^; 1^f^ | 1^e^; 1^f^ | 1^e^; 1^f^ | 2^e^; 2^f^ | 2^e^; 2^f^ | 2^e^; 2^f^ | 1^e^; 1^f^ | 0^e^; 1^f^ | 1^e^; 1^f^ | 0^e^; 1^f^ | 0^e^; 0^f^ | 1^e^; 1^f^ | 1^e^; 1^f^ | 0^e^; 0^f^ | 1^e^; 1^f^ |
| 9 | 0^e^; 0^f^ | 0^e^; 0^f^ | 0^e^; 0^f^ | 0^e^; 0^f^ | 0^e^; 0^f^ | 0^e^; 0^f^ | 0^e^; 0^f^ | 0^e^; 0^f^ | 0^e^; 0^f^ | 3^e^; 1^f^ | 1^e^; 1^f^ | 1^e^; 1^f^ | 1^e^; 1^f^ | 1^e^; 1^f^ | 1^e^; 1^f^ | 1^e^; 1^f^ | 1^e^; 1^f^ | 1^e^; 1^f^ | 1^e^; 1^f^ | 1^e^; 1^f^ | 1^e^; 1^f^ |
| 10 | 5^e^; 5^f^ | 5^e^; 5^f^ | 5^e^; 5^f^ | 1^e^; 1^f^ | 1^e^; 1^f^ | 1^e^; 1^f^ | 1^e^; 1^f^ | 1^e^; 1^f^ | 1^e^; 1^f^ | 0^e^; 0^f^ | 0^e^; 0^f^ | 0^e^; 0^f^ | 1^e^; 1^f^ | 1^e^; 1^f^ | 1^e^; 1^f^ | 0^e^; 1^f^ | 0^e^; 1^f^ | 1^e^; 1^f^ | 1^e^; 1^f^ | 1^e^; 1^f^ | 1^e^; 1^f^ |
| 11 | 1^e^; 1^f^ | 1^e^; 1^f^ | 1^e^; 1^f^ | 0^e^; 0^f^ | 0^e^; 0^f^ | 0^e^; 0^f^ | 0^e^; 0^f^ | 0^e^; 0^f^ | 0^e^; 0^f^ | 2^e^; 2^f^ | 2^e^; 2^f^ | 2^e^; 2^f^ | 1^e^; 1^f^ | 1^e^; 1^f^ | 1^e^; 1^f^ | 1^e^; 1^f^ | 1^e^; 1^f^ | 1^e^; 1^f^ | 1^e^; 1^f^ | 1^e^; 1^f^ | 1^e^; 1^f^ |
| 12 | 0^e^; 0^f^ | 0^e^; 0^f^ | 0^e^; 0^f^ | 0^e^; 0^f^ | 0^e^; 0^f^ | 0^e^; 0^f^ | 0^e^; 0^f^ | 0^e^; 0^f^ | 0^e^; 0^f^ | 1^e^; 1^f^ | 1^e^; 1^f^ | 1^e^; 1^f^ | 1^e^; 1^f^ | 1^e^; 1^f^ | 1^e^; 1^f^ | 0^e^; 0^f^ | 1^e^; 1^f^ | 1^e^; 1^f^ | 0^e^; 0^f^ | 1^e^; 1^f^ | 1^e^; 1^f^ |
| 13 | 1^e^; 1^f^ | 1^e^; 1^f^ | 1^e^; 1^f^ | 1^e^; 1^f^ | 1^e^; 1^f^ | 1^e^; 1^f^ | 0^e^; 0^f^ | 0^e^; 0^f^ | 0^e^; 0^f^ | 0^e^; 0^f^ | 0^e^; 0^f^ | 0^e^; 0^f^ | 1^e^; 1^f^ | 1^e^; 1^f^ | 1^e^; 1^f^ | 1^e^; 1^f^ | 1^e^; 1^f^ | 1^e^; 1^f^ | 0^e^; 0^f^ | 1^e^; 1^f^ | 0^e^; 0^f^ |
| 14 | 2^e^; 2^f^ | 1^e^; 1^f^ | 2^e^; 1^f^ | 2^e^; 2^f^ | 2^e^; 2^f^ | 2^e^; 2^f^ | 1^e^; 1^f^ | 1^e^; 1^f^ | 1^e^; 1^f^ | 0^e^; 0^f^ | 0^e^; 0^f^ | 0^e^; 0^f^ | 1^e^; 1^f^ | 1^e^; 1^f^ | 1^e^; 1^f^ | 0^e^; 0^f^ | 1^e^; 1^f^ | 1^e^; 1^f^ | 0^e^; 0^f^ | 1^e^; 1^f^ | 1^e^; 1^f^ |
| 15 | 0^e^; 0^f^ | 0^e^; 0^f^ | 0^e^; 0^f^ | 1^e^; 1^f^ | 1^e^; 1^f^ | 1^e^; 1^f^ | 0^e^; 0^f^ | 0^e^; 0^f^ | 0^e^; 0^f^ | 3^e^; 3^f^ | 3^e^; 3^f^ | 3^e^; 3^f^ | 1^e^; 1^f^ | 1^e^; 1^f^ | 1^e^; 1^f^ | 0^e^; 0^f^ | 1^e^; 1^f^ | 1^e^; 1^f^ | 0^e^; 0^f^ | 1^e^; 1^f^ | 1^e^; 1^f^ |
| 16 | 2^e^; 2^f^ | 2^e^; 2^f^ | 2^e^; 2^f^ | 2^e^; 2^f^ | 2^e^; 2^f^ | 2^e^; 2^f^ | 1^e^; 1^f^ | 1^e^; 1^f^ | 1^e^; 1^f^ | 0^e^; 0^f^ | 0^e^; 0^f^ | 0^e^; 0^f^ | 0^e^; 0^f^ | 1^e^; 0^f^ | 0^e^; 0^f^ | 0^e^; 0^f^ | 1^e^; 0^f^ | 0^e^; 0^f^ | 0^e^; 0^f^ | 0^e^; 0^f^ | 0^e^; 0^f^ |
| 17 | 3^e^; 5^f^ | 3^e^; 5^f^ | 3^e^; 5^f^ | 1^e^; 1^f^ | 1^e^; 1^f^ | 1^e^; 1^f^ | 1^e^; 1^f^ | 1^e^; 1^f^ | 1^e^; 1^f^ | 0^e^; 0^f^ | 0^e^; 0^f^ | 0^e^; 0^f^ | 0^e^; 0^f^ | 0^e^; 0^f^ | 0^e^; 0^f^ | 1^e^; 1^f^ | 1^e^; 1^f^ | 1^e^; 1^f^ | 1^e^; 1^f^ | 1^e^; 1^f^ | 1^e^; 1^f^ |
| 18 | 1^e^; 1^f^ | 1^e^; 1^f^ | 1^e^; 1^f^ | 1^e^; 2^f^ | 1^e^; 2^f^ | 1^e^; 1^f^ | 0^e^; 1^f^ | 0^e^; 1^f^ | 0^e^; 0^f^ | 0^e^; 0^f^ | 0^e^; 0^f^ | 0^e^; 0^f^ | 1^e^; 1^f^ | 1^e^; 1^f^ | 1^e^; 1^f^ | 1^e^; 1^f^ | 0^e^; 0^f^ | 1^e^; 1^f^ | 0^e^; 0^f^ | 0^e^; 0^f^ | 0^e^; 0^f^ |
| 19 | 0^e^; 0^f^ | 0^e^; 0^f^ | 0^e^; 0^f^ | 0^e^; 0^f^ | 0^e^; 0^f^ | 0^e^; 0^f^ | 0^e^; 0^f^ | 0^e^; 0^f^ | 0^e^; 0^f^ | 1^e^; 1^f^ | 1^e^; 1^f^ | 1^e^; 1^f^ | 1^e^; 1^f^ | 1^e^; 1^f^ | 1^e^; 1^f^ | 1^e^; 1^f^ | 1^e^; 1^f^ | 1^e^; 1^f^ | 1^e^; 1^f^ | 1^e^; 1^f^ | 1^e^; 1^f^ |
| 20 | 2^e^; 3^f^ | 2^e^; 2^f^ | 2^e^; 2^f^ | 0^e^; 0^f^ | 0^e^; 0^f^ | 0^e^; 0^f^ | 1^e^; 1^f^ | 1^e^; 1^f^ | 1^e^; 1^f^ | 2^e^; 2^f^ | 2^e^; 2^f^ | 2^e^; 2^f^ | 1^e^; 1^f^ | 1^e^; 1^f^ | 1^e^; 1^f^ | 0^e^; 1^f^ | 1^e^; 1^f^ | 0^e^; 0^f^ | 1^e^; 1^f^ | 1^e^; 1^f^ | 1^e^; 1^f^ |
| 21 | 0^e^; 0^f^ | 0^e^; 0^f^ | 0^e^; 0^f^ | 0^e^; 0^f^ | 0^e^; 0^f^ | 0^e^; 0^f^ | 0^e^; 0^f^ | 0^e^; 0^f^ | 0^e^; 0^f^ | 2^e^; 2^f^ | 2^e^; 2^f^ | 2^e^; 2^f^ | 1^e^; 1^f^ | 1^e^; 1^f^ | 1^e^; 1^f^ | 1^e^; 1^f^ | 1^e^; 1^f^ | 1^e^; 1^f^ | 1^e^; 1^f^ | 1^e^; 1^f^ | 1^e^; 1^f^ |
| 22 | 1^e^; 1^f^ | 1^e^; 1^f^ | 1^e^; 1^f^ | 1^e^; 1^f^ | 1^e^; 1^f^ | 1^e^; 1^f^ | 0^e^; 0^f^ | 0^e^; 0^f^ | 0^e^; 0^f^ | 0^e^; 0^f^ | 0^e^; 0^f^ | 0^e^; 0^f^ | 1^e^; 1^f^ | 0^e^; 0^f^ | 1^e^; 1^f^ | 1^e^; 1^f^ | 0^e^; 0^f^ | 1^e^; 1^f^ | 1^e^; 1^f^ | 1^e^; 0^f^ | 1^e^; 1^f^ |
| 23 | 1^e^; 1^f^ | 1^e^; 1^f^ | 1^e^; 1^f^ | 1^e^; 1^f^ | 1^e^; 1^f^ | 1^e^; 1^f^ | 0^e^; 0^f^ | 0^e^; 0^f^ | 0^e^; 0^f^ | 0^e^; 0^f^ | 0^e^; 0^f^ | 0^e^; 0^f^ | 1^e^; 1^f^ | 1^e^; 1^f^ | 1^e^; 1^f^ | 0^e^; 0^f^ | 1^e^; 1^f^ | 1^e^; 1^f^ | 0^e^; 0^f^ | 1^e^; 1^f^ | 1^e^; 1^f^ |

^a^ 0: grade 0 (no extension of the medial carotid line); 1: grade 1 (extension of the medial line, but no extension of the intercarotid line); 2: grade 2 (extension of the intercarotid line, but no extension of the lateral line); 3: grade 3A (extension of the lateral line superior to the intracavernous carotid artery); 4: grade 3B (extension of the lateral line inferior to the intracavernous carotid artery); and 5: grade 4 (intracavernous carotid artery totally surrounded) (Supplementary Fig. 1) [23,24].

^b^ 0: not invasive; 1: invasive [23]

^c^ 0: not visible; 1: lateral ipsilateral side; 2: lateral contralateral side; 3: superior [23]

^d^ 0: not visible; 1: visible [23]

^e^ Assessment performed by observer 1

^f^ Assessment performed by observer 2

a: Contrast-enhanced, MDME sequence-based T1-weighted MR imaging data

b: Contrast-enhanced, VIBE sequence-based T1-weighted MR imaging data

c: Contrast-enhanced, MPRAGE sequence-based T1-weighted MR imaging data

CN: Cranial nerve
