# Supplementary figures and images for: Multi-Dynamic-Multi-Echo-based MRI for the Pre-Surgical Determination of Sellar Tumor Consistency: a Quantitative Approach for Predicting Lesion Resectability
Source: Clin Neuroradiol. 2024 Apr 19;34(3):663–73. doi: 10.1007/s00062-024-01407-1 (PMC11339083; doi:10.1007/s00062-024-01407-1)

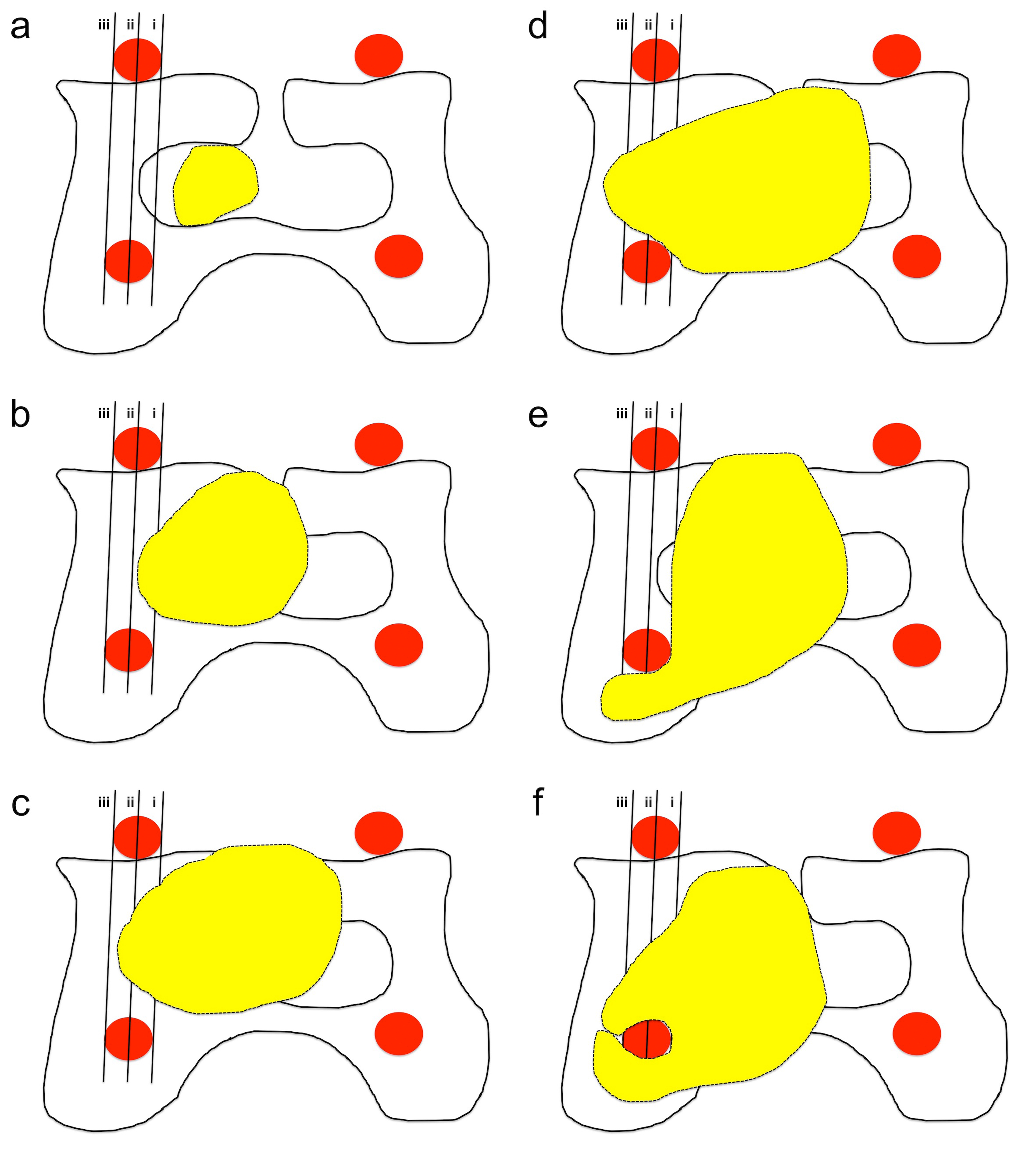

Supplement: Supplementary file 3 — Supplementary Table 1: Quantitative Magnetic Resonance Imaging Metrics Determined by Both Raters [file 62_2024_1407_MOESM3_ESM.jpg]

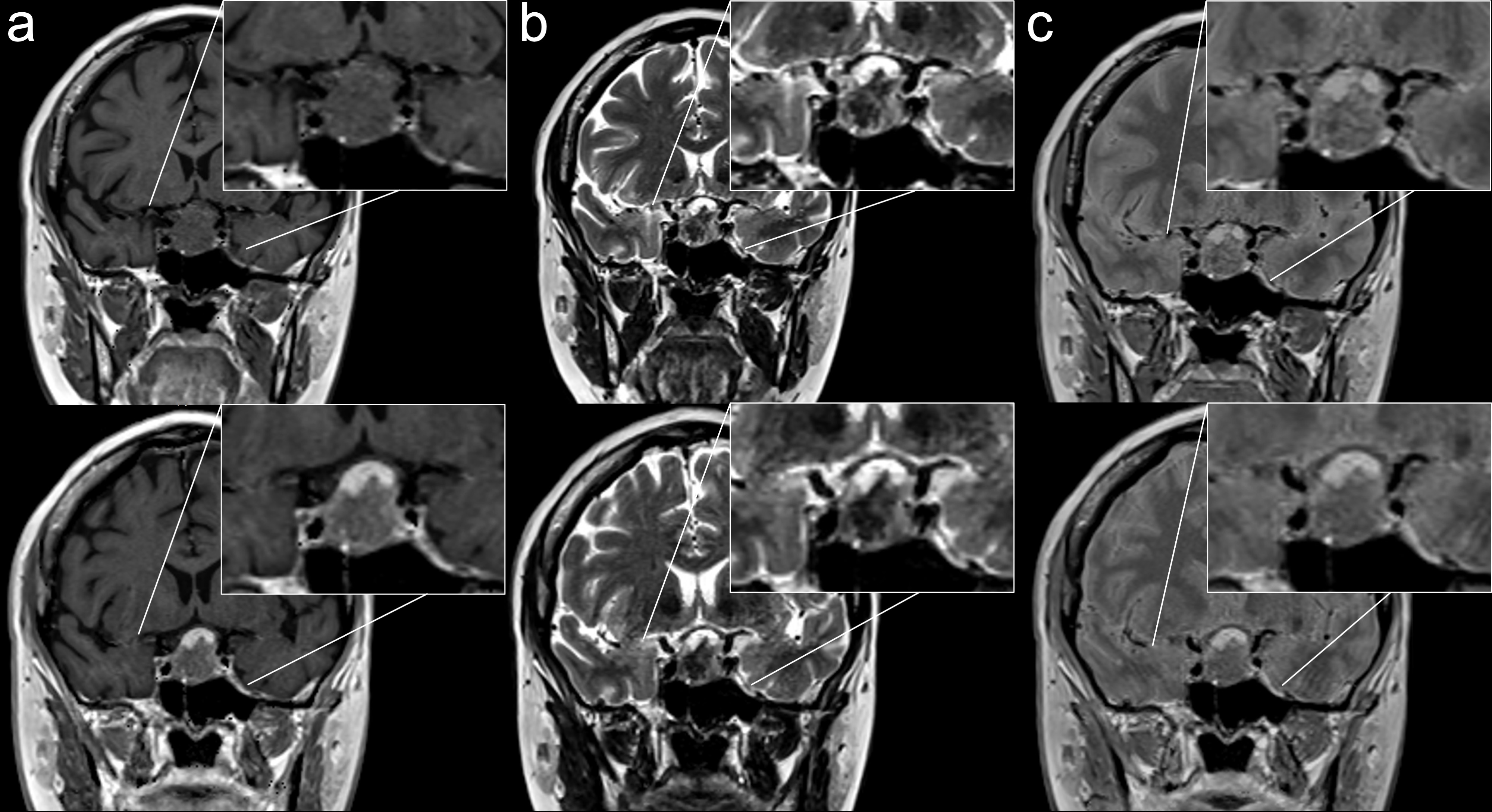

Supplement: Supplementary file 4 — Supplementary Table 2: Qualitative Assessments of the pituitary Region by both raters [file 62_2024_1407_MOESM4_ESM.jpg]
